# A Randomized, Double-Blinded, Placebo-Controlled, Clinical Study of the Effects of a Nutraceutical Combination (LEVELIP DUO^®^) on LDL Cholesterol Levels and Lipid Pattern in Subjects with Sub-Optimal Blood Cholesterol Levels (NATCOL Study)

**DOI:** 10.3390/nu12103127

**Published:** 2020-10-14

**Authors:** Arrigo F.G. Cicero, Sergio D’Addato, Claudio Borghi

**Affiliations:** Medical an Surgery Sciences Department, Dyslipidemia and Atherosclerosis Research Unit, Alma Mater Studiorum University of Bologna, 40138 Bologna, Italy; sergio.daddato@unibo.it (S.D.); claudio.borghi@unibo.it (C.B.)

**Keywords:** monacolins, LDL-cholesterol, phytosterols, red yeast rice, nutraceuticals, clinical trial, endothelial function

## Abstract

Phytosterols and red yeast rice are largely studied cholesterol-lowering nutraceuticals, respectively inhibiting the bowel absorption and liver synthesis of cholesterol. Our aim was to test the effect of combined nutraceutical-containing phytosterols and red yeast rice vs. a placebo on the lipid profile. We performed a parallel arms, double-blind, placebo-controlled clinical trial, randomizing 88 moderately hypercholesterolemic subjects to treatment with a combined nutraceutical containing phytosterols (800 mg) and red yeast rice, standardized to contain 5 mg of monacolins from *Monascus purpureus*, with added niacin (27 mg) and policosanols (10 mg) (LEVELIP DUO^®^)*,* or placebo. The mean LDL-Cholesterol (LDL-C) change at Week 8 was −32.5 ± 30.2 mg/dL (−19.8%) in the combined nutraceutical group and 2.5 ± 19.4 mg/dL (2.3%) in the placebo group. The estimated between-group difference of −39.2 mg/dL (95% CI: −48.6; −29.8) indicates a statistically significant difference between treatments in favor of the combined nutraceutical (*p* < 0.0001). Total Cholesterol (TC), non-HDL cholesterol (non-HDL-C), Apolipoprotein B, TC/HDL-C and LDL-C/HDL-C improved in a similar way in the combined nutraceutical group only. No significant changes in other clinical and laboratory parameters were observed. In conclusion, the tested combined nutraceutical was well tolerated, while significantly reducing the plasma levels of LDL-C, TC, non-HDL-C, ApoB, TC/HDL-C and LDL-C/HDL-C ratios in mildly hypercholesterolemic patients. Trial registration (ClinicalTrials.gov): NCT03739242.

## 1. Introduction

Hypercholesterolemia is a largely prevalent cardiovascular disease risk factor in the general population, and its early reduction seems to be an effective preventive strategy [1]. However, pharmacologically treating moderately hypercholesterolemic subjects without other cardiovascular risk factors in the primary prevention for cardiovascular disease is still debated [2]. In fact, the guidelines suggest managing these subjects with a more conservative approach, stressing the need for a therapeutic lifestyle promotion, eventually added with lipid-lowering nutraceuticals and/or functional foods [1,3].

In the last few decades, a relatively large number of nutraceuticals and functional foods has been studied for their ability to decrease cholesterolemia in humans [4]. The most clinically studied include soluble fibers, phytosterols, soy proteins, monacolins from red yeast rice, berberine, and garlic and artichoke extracts [5]. In particular, the most recent guidelines for dyslipidaemia management from the European Atherosclerosis Society and from the European Cardiology Society suggest increasing the amounts of fiber and omega 3 polyunsaturated fatty acids in the diet, while adding phytosterols and monacolins as dietary supplements, when a lifestyle intervention is needed to reduce cholesterolemia [6].

Plant sterols and stanols (phytosterols) are natural constituents of the plant cell membrane [7]. From a chemical point of view, they are very similar to cholesterol, with minor differences in the relative positions of ethyl and methyl groups. Based on this similarity, phytosterols may compete with dietary and biliary cholesterol for micellar solubilization in the intestinal lumen, impairing intestinal cholesterol absorption [8]. Moreover, several clinical trials have consistently shown that an intake of 2–3 g/day of plant sterols is associated with a significant lowering (between 4% and 15%) of low-density lipoprotein-cholesterol (LDL-C) [9,10]. The variability in the observed LDL-reduction is mainly related to genetic factors [11]. Based on the available data, the European Food Safety Agency (EFSA) accepted a health claim for the phytosterols’ LDL-C-lowering effect [12].

Red yeast rice is a nutraceutical obtained by the fermentation of rice (*Oryza sativa*) as result of a yeast (in general *Monascus purpureus*), whose typical red coloration is due to the presence of some specific pigments, by-products of the fermentative metabolism process [13]. Monascus yeast produces a family of substances called monacolins, including monacolin K. Monacolins act as reversible inhibitors of the 3-hydroxy-3-methyl-glutaryl-coenzyme A reductase, the key enzyme in cholesterol biosynthesis [14].

A recent meta-analysis of 20 randomized clinical trials, including 6663 subjects, showed that, after 2–24 months of treatment, red yeast rice (RYR) reduced LDL-C on average by 39.4 mg/dL compared to placebo, which was comparable to the reduction achieved with regular-dosed statins (pravastatin 40 mg, simvastatin 10 mg, lovastatin 20 mg) [15]. Based on these data, the EFSA has expressed a scientific opinion supporting the health claims for the relationship between the administration of RYR and the control of plasma LDL-C levels [16]. Even if recently the same EFSA raised some concerns about RYR’s safety [17], a recent large metanalysis of 53 randomized clinical trials, including 8535 subjects, has shown that monacolin K administration is not associated with an increased risk of Statin Associated Muscle Symptoms (SAMS) (odds ratio (OR) = 0.94, 95% confidence interval (CI) 0.53, 1.65) for daily doses of monacolin K of between 3 and 10 mg [18].

In this context, the primary objective of the study was to evaluate the effects of LEVELIP DUO^®^ on LDL-C blood levels in subjects with sub-optimal blood cholesterol levels over an 8-week period. The secondary objective was the evaluation of effects of the tested combined nutraceuticals on other lipoproteins and on the estimated cardiovascular risk.

## 2. Materials and Methods

This parallel-armed, double-blind, randomized clinical trial was carried out in 90 moderately hypercholesterolemic subjects, non-smokers, pharmacologically untreated, in primary prevention for cardiovascular diseases, consecutively enrolled in the ambulatory service of cardiovascular disease prevention in the Medical and Surgical Sciences Department of the University of Bologna.

The inclusion criteria were age between 30 and 75, and LDL-C level between 115 and 190 mg/dL, confirmed in at least two sequential checks prior to signing the consent form.

The exclusion criteria were as follows:

- Personal history of cardiovascular disease or risk equivalents;
- Triglycerides (TG) ≥400 mg/dL;
- Obesity (Body Mass Index > 32 kg/m^2^);
- Assumption of lipid-lowering drugs or supplements affecting lipid metabolism;
- Uncontrolled diabetes mellitus;
- Known thyroid, liver, renal or muscle diseases.

The study was fully conducted in accordance with the Declaration of Helsinki, its protocol was approved by the Ethical Committee of the University of Bologna, and informed consent was obtained from all patients before inclusion in the study (Clinical trial.gov ID NCT03739242).

**Figure 1:**
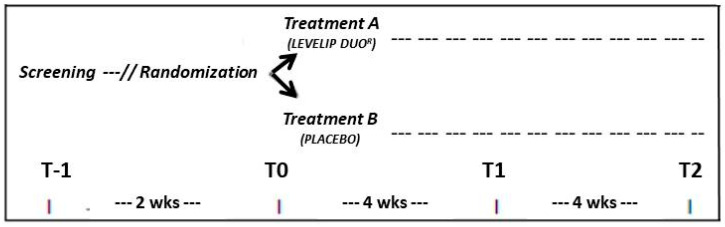
Study design.

The study design has been detailed in Figure 1.

At the enrollment visit (T-1, Day-14), patients were given standard behavioral and qualitative (not quantitative) dietary suggestions to correct unhealthy habits. Standard diet advice was given by a dietitian and/or specialist doctor. A dietitian and/or specialist doctor periodically provided instructions on dietary intake recording procedures as part of a behavior modification program, and then later used the subject’s food diaries for counseling. In particular, the subjects were instructed to follow the general structure of a Mediterranean diet, avoid excessive intakes of dairy products and red meat-derived products during the study, and maintain overall constant dietary habits [19]. Individuals were also generically encouraged to increase their physical activity by walking briskly for 20 to 30 min, 3 to 5 times per week, or by cycling [20].

After 2 weeks of diet and physical activity (Randomization visit, T0), if the LDL-C and TG values were confirmed, the patients were randomly allocated to a treatment with LEVELIP DUO^®^ or placebo, one tablet after dinner. LEVELIP DUO^®^ is a registered combination of phytosterols (800 mg) and other registered ingredients including red yeast rice standardized to contain 5 mg monacolins from *Monascus purpureusi,* with niacin (27 mg) and policosanols (10 mg) (Dif1/Stat^®^) [21,22]. The red yeast rice extract used was certified to be highly purified in monacolins, without chromatographically detectable levels of dehydromonacolins, decalin derivatives and contaminants. The phytosterol dose was chosen based on the minimal efficacious dose identified by the meta-analysis of randomized clinical trials, performed by Demonty et al. [23].

The active products and placebo were administered as indistinguishable tablets (kindly provided by Menarini IFR, Firenze, Italy). The treatment then continued for 8 weeks. Clinical and laboratory data have been obtained at the baseline (T0), after 4 weeks (T1) and at the end of the trial (8 weeks, T2).

A computer-generated randomization list produced by the trial statistician randomized patients to one of the two treatment groups (LEVELIP DUO^®^ or placebo) in a 1:1 ratio. A paper-based randomization procedure assigned a randomization number to the patient, which has been used to link the patient to a treatment arm and specified a unique kit number for the package of treatment to be dispensed to the patient. The randomization numbers were generated using a procedure to ensure that treatment assignment is unbiased.

Throughout the study, we instructed patients to take the first dose on the day after they were given the study product in a blinded box. At the end of the study, all unused products were retrieved for inventory. Product compliance was assessed by counting the number of product doses returned at the times of specified clinic visits.

At each visit, enrolled patients were interviewed about eventual changes in lifestyle and possible adverse events raised in the previous weeks. Then, anthropometric measurement and vital signs were recorded, and a plasma sample was obtained after a 12-h overnight fast. Venous blood samples were drawn by a nurse from all patients between 8:00 a.m. and 9:00 a.m. The serum used was centrifuged at 3000 g for 15 min at ambient temperature. Immediately after centrifugation, the samples were frozen and stored at −80 °C for no more than 3 months. The following parameters were evaluated via standardized methods [24,25]: total cholesterol (TC), HDL-C, TG, apolipoprotein B100 (apoB), glucose, creatinine, serum uric acid, liver transaminases, gamma-glutamyl transferase, and creatinine phosphokinase (CPK). All measurements were performed by trained personnel in the Lipid Clinic laboratory of the Medicine and Surgery Sciences Department, by the S. Orsola-Malpighi University Hospital. Since hypertriglyceridemia was an exclusion criterion, the LDL-C level was estimated by the application of the Friedewald’s formula (LDL-C = TC − HDL-C − TG/5).

As post-hoc analysis, the cardiovascular disease risk was estimated with a validated nation-specific algorithm (Progetto CUORE) [26].

Considering the primary endpoint of the study as the reduction from baseline to week 8 in LDL cholesterol level, and the data available from literature [27], a reduction in LDL level of approximately 10% is expected after the intake of the nutraceutical. Therefore, assuming a baseline LDL level of 145 ± 19 mg/dL, a power of 90% and a 5% two-sided alpha level to detect a difference in mean change in LDL from baseline to week 8 equal to 15 mg/dL between the nutraceutical and the placebo group, the total number of patients to be evaluated should be 35 per treatment arm in a 1:1 ratio (NQuery Advisor, 7.0). Allowing for an approximate 20% dropout rate, at least 88 patients should be randomized—44 patients in each treatment group.

Statistical tables, figures, listings and analyses were produced using SAS^®^ for Windows release 9.4 (64-bit) or later (SAS Institute Inc., Cary, NC, USA). For each secondary efficacy variable, an ANCOVA model was used to estimate the treatment’s effect on the changes from baseline at week 8, considering the planned treatment group as the factor and the baseline value of the parameter as the continuous covariate. The results are reported as Least Squares Means together with associated two-tailed 95% CI. The difference in Least Squares Means between the nutraceutical combination group and placebo group was estimated with two-tailed 95% CI and *p* value. If the assumption of the normality of the residuals was violated, the ANCOVA model was fitted to rank transformed data. A *p* value less than 0.05 was considered significant for all tests.

## 3. Results

**Figure 2:**
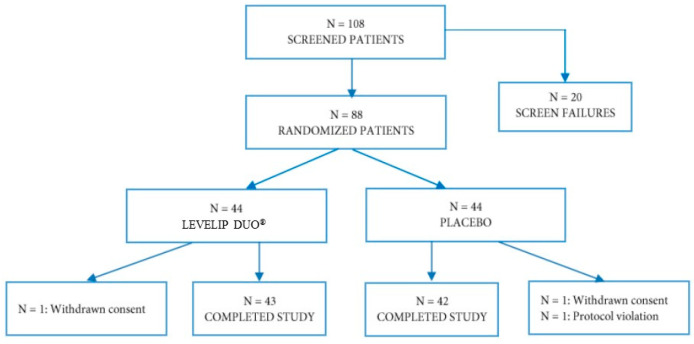
Patient disposition.

Enrolled patients were age- and sex-matched. We enrolled 38 men (19 randomized to the combined nutraceutical, 19 to placebo) and 47 women (24 randomized to the combined nutraceutical, 23 to placebo). The baseline characteristics of patients assigned to the different treatments (active and placebo) were similar, and no significant differences were observed regarding the studied parameters (Table 1). Patient disposition is outlined in Figure 2.

Overal, 85 subjects (94.4%), 43 in the active treatment group and 42 in the placebo group, were compliant (compliance levels between 80% and 120%). One patient (2.33%) in the active treatment group had compliance lower than 80%, whereas 1 patient (2.33%) in the placebo group had compliance greater than 120%.

Dietary habits remained overall unchanged during the study.

**Figure 3:**
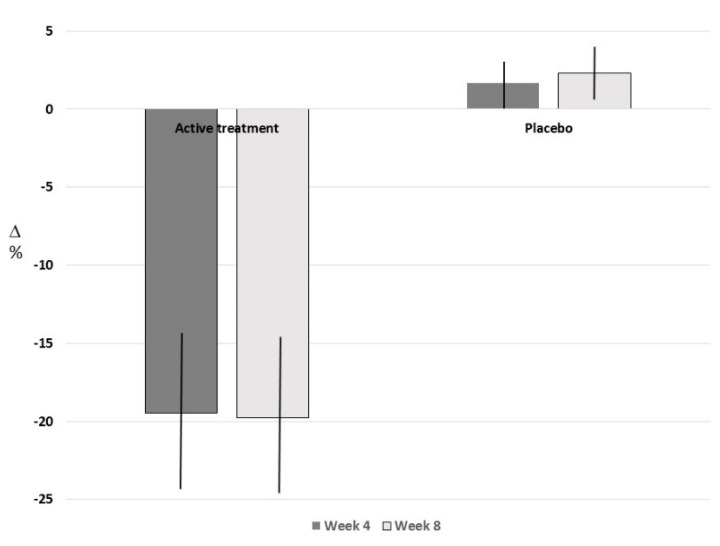
Plasma LDL-Cholesterol level percentage change from the baseline to 4 and 8 weeks in subjects treated with the tested combined nutraceutical or placebo.

The primary variable of the study was the change from baseline at week 8 in LDL cholesterol (Figure 3).

The LDL-C levels decreased from visit to visit in the combined nutraceutical group, and, on the contrary, increased in the placebo group. The mean change at week 8 was −32.5 ± 30.2 mg/dL (−19.8%) in the combined nutraceutical group and 2.5 ± 19.4 mg/dL (2.3%) in the placebo group. The ANCOVA model results for the estimated change from baseline at week 8 in LDL-C levels, adjusting for the baseline value of LDL-C, provided Least Squares Means equal to −34.5 mg/dL (95% CI: −41.1; −27.9) for the combined nutraceutical group and 4.6 mg/dL (95% CI: −2.0; 11.3) for the placebo group. The estimated between-group difference of −39.2 mg/dL (95% CI: −48.6; −29.8) indicates a statistically significant difference between treatments in favor of the combined nutraceutical (*p* < 0.0001).

The Least Squares Means of TC changes from baseline at week 8, adjusting for baseline value, were −33.0 mg/dL (95% CI: −40.4; −25.7) for the combined nutraceutical group and 8.2 mg/dL (95% CI: 0.8; 15.7) for the placebo group. The estimated between-group difference of −41.2 mg/dL (95% CI: −51.7; −30.7) and a *p* < 0.0001 suggest a statistically significant difference between treatments in favor of the combined nutraceutical.

The Least Squares Means of non-HDL cholesterol change from baseline at week 8, estimated adjusting for baseline value, were equal to −34.2 mg/dL (95% CI: −41.1; −27.3) in the combined nutraceutical group and 7.5 mg/dL (95% CI: 0.5; 14.5) in the placebo group. The between-group difference of −41.7 mg/dL (95% CI: −51.6; −31.8) was significantly in favor of treatment with the combined nutraceutical (*p* < 0.0001).

The Least Squares Means of Apo B change at week 8, adjusting for baseline value, were −15.2 mg/dL (95% CI: −19.6; −10.8) for the combined nutraceutical group and 2.0 mg/dL (95% CI: −2.4; 6.5) for placebo. The between-group difference of −17.3 mg/dL (95% CI: −23.5; −11.0) indicates a statistically significant difference between treatments in favor of the combined nutraceutical (*p* < 0.0001).

The Least Squares Means of the TC/HDL-C ratio change from baseline at week 8, adjusting for baseline value, were −0.8 (95% CI: −1.0; −0.6) for the combined nutraceutical group and 0.1 (95% CI: −0.1; 0.3) for placebo. The estimated between-group difference was −0.9 (95% CI: −1.2; −0.6), and a *p* < 0.0001 indicates a significantly greater change in the TC/HDL ratio in subjects treated with the combined nutraceutical.

The Least Squares Means of the LDL-C/HDL-C ratio change from baseline at week 8, adjusting for baseline value, were −0.8 (95% CI: −0.9; −0.6) for the combined nutraceutical group and 0.02 (95% CI: −0.1; 0.2) for the placebo group. The between-group difference of −0.8 (95% CI: −1.1; −0.6) was statistically significant (*p* < 0.0001).

Body weight, BMI, waist circumference, blood pressure, FPG, HDL-C, TG, GOT, GPT, gGT, SUA, eGFR and CPK did not significantly change in both groups during the study (Table 2).

**Figure 4:**
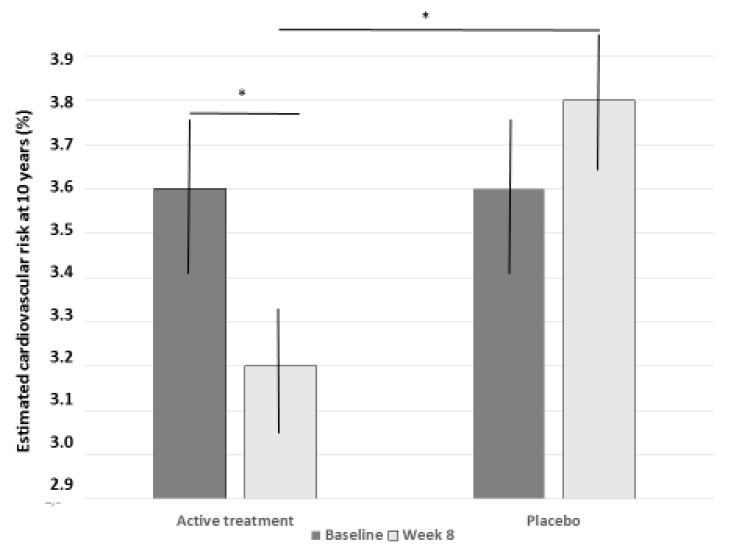
Change in estimated 10-year cardiovascular disease risk in the two treatment groups (post-hoc analysis; * *p* < 0.05).

The estimated cardiovascular risk significantly decreased in the combined nutraceutical-treated subjects (*p* < 0.01), while it remained unchanged in the placebo group (Figure 4).

During the study, no treatment-emergent adverse event was reported. The trends of hematology and clinical chemistry values did not indicate any safety concerns.

## 4. Discussion

The Mediterranean diet remains a milestone in cardiovascular disease prevention, [28] even if its impact on LDL-cholesterolemia is limited. For this reason, the ESC/EAS guidelines [6] and the International Lipid Expert Panel (ILEP) [5] consider the use of some dietary supplements (namely, red yeast rice and phytosterols) as a support to a balanced diet in order to improve cholesterolemia control. Many trials evaluating the effect of nutraceuticals on lipid pattern are not adequately designed, being often not double-blinded and underpowered.

In our double-blind, placebo-controlled, randomized clinical trial, we observed that the LEVELIP DUO^®^ was able in the short-term to significantly reduce the plasma levels of LDL-C, TC, non-HDL-C, and apoB, and the TC/HDL-C and LDL-C/HDL-C ratios. In particular, the estimated between-group difference in LDL-C was −39.2 mg/dL (95% CI: −48.6; −29.8). This effect was observed after 4 weeks of treatment and confirmed after 8 weeks, excluding short-term adaptation phenomena. This is compatible with the mechanism of action of the nutraceutical components of the tested product. The LDL-C reduction that was achieved is near to that 39.8 mg/dL, which is estimated to be associated in long-term trials with a corresponding 22% reduction in cardiovascular mortality and morbidity [29]. This is also in line with our observation that the estimated cardiovascular risk was significantly modified by the tested treatment, but not by the placebo. This impressive result was partly expected. In fact, it confirms what we already observed in a previous smaller pilot study, where the association of phytosterols and red yeast rice was able to increase red yeast rice’s LDL-C-lowering efficacy [30]. On the other side, because the mechanisms of action of red yeast rice and phytosterols should be additive or synergistic, they represent the natural alternative to the synergistic association of statins with ezetimibe [27].

In this context, the tested combined nutraceutical was shown to be effective and well-tolerated. In 2017, the ILEP [5] classified the association of phytosterols and red yeast rice as recommendation IIa (should be considered), based on a level of evidence classified as B (Data derived from single randomized clinical trial or large non-randomized studies). Considering the results of our current trial and of our previous one [31], combined with the ESC/EAS suggestion, the class of recommendation could probably improve.

We can argue that the greater part of the observed effect of LEVELIP DUO^®^ is be related to the contents of phytosterols and red yeast rice. However, the minor components of the products could have also minimally contributed to the final effect. In particular, EFSA approves the health claim of niacin supporting energy metabolism and macronutrient metabolism [32]. Policosanols seem to have a small impact on hypercholesterolemia, however their efficacy when consumed with red yeast rice has been observed in a large number of trials [5].

Our study has some relevant limitations. The first one is the relatively low number of subjects investigated per treatment group, while, however, the study was sufficiently powered to detect differences between treatment groups. Of course, we could have used a cross-over design, but we feared losing the volunteers’ compliance to the treatment if the study duration was excessive. The second one is the lack of the measurement of markers of cholesterol absorption and synthesis, as cholesterol hyperabsorbers could have manifested a more significant LDL-C reduction than standard cholesterol absorbers [31]. Finally, the study was relatively short, so that we do not know if the observed effect could be confirmed in the long term. However, since the monacolins’ and phytosterols’ mechanisms of action are the same as those of statins and ezetimibe, drugs that have largely proven to maintain their efficacy over decades, we could reasonably assume that this evidence could be translated to the tested combined nutraceutical. Moreover, the study was adequately powered, so that we can be confident in the reported results.

## 5. Conclusions

LEVELIP DUO^®^ was well-tolerated while significantly reducing the plasma levels of LDL-C, TC, non-HDL-C, ApoB, TC/HDL-C and LDL/HDL-C ratios in mildly hypercholesterolemic patients. Further long-term studies are needed to confirm the maintenance of this impressive effect over time.

## Figures and Tables

**Table 1:** Baseline characteristics of the enrolled subjects: non-statistically significant difference has been detected between the treatment groups (values reported as mean ± standard deviation; No significant difference between groups has been observed).

	Active Treatment	Placebo
Age (years)	51.3 ± 9.6	51.8 ± 10.7
Height (m)	1.69 ± 0.09	1.69 ± 0.10
Weight (kg)	70.3 ± 12.0	71.2 ± 14.8
Body Mass Index (kg/m^2^)	24.5 ± 3.2	24.7 ± 3.1
Waist circumference (cm)	89.5 ± 10.6	91.6 ± 11.1
Systolic Blood Pressure (mmHg)	124 ± 16	124 ± 15
Diastolic Blood Pressure (mmHg)	77 ± 10	78 ± 9
Heart Rate (bpm)	70 ± 10	70 ± 9
Total cholesterol (mg/dL)	229.1 ± 27.9	232.6 ± 21.6
LDL cholesterol (mg/dL)	155.1 ± 19.9	161.5 ± 21.3
HDL cholesterol (mg/dL)	51.0 ± 13.5	49.0 ± 11.3
Non-HDL cholesterol (mg/dL)	178.1 ± 25.5	183.6 ± 22.2
Triglycerides (mg/dL)	114.9 ± 46.2	110.4 ± 46.3
Apolipoprotein B (mg/dL)	106.9 ± 13.6	110.8 ± 15.8
TC/HDL-Cholesterol	4.76 ± 1.22	4.98 ± 1.19
LDL-C/HDL-Cholesterol	3.25 ± 0.99	3.48 ± 0.93
Fasting Glucose (mg/dL)	89.1 ± 11.6	88.8 ± 11.4
Alanine aminotransferase (U/L)	22.5 ± 8.6	23.4 ± 10.3
Aspartate aminotransferase (U/L)	20.9 ± 5.6	21.7 ± 4.0
Gamma-glutamyl transferase (U/L)	22.1 ± 13.6	21.2 ± 13.1
Serum uric acid (mg/dL)	4.4 ± 1.3	4.2 ± 0.9
Serum creatinine (mg/dL)	0.94 ± 0.13	0.96 ± 0.16
Estimated GFR (ml/min/1.73 m^2^)	77.3 ± 8.3	76.1 ± 11.9
Creatine phosphokinase (U/L)	110 ± 65	131 ± 77

**Table 2:** Laboratory parameter changes during the study (values reported as mean ± standard deviation).

Variable	Active Treatment			Placebo		
	Baseline (T0)	Day 28 (T1)	Day 56 (T2)	Baseline (T0)	Day 28 (T1)	Day 56 (T2)
TC (mg/dL)	229.1 ± 27.9	196.7 ± 24.1 * °	197.1 ± 28.3 * °	232.6 ± 21.6	237.5 ± 23.6	239.8 ± 23.6
LDL-C (mg/dL)	155.1 ± 19.9	124.0 ± 22.3 * °	122.6 ± 24.7 * °	161.5 ± 21.3	163.3 ± 22.3	164.0 ± 20.4
HDL-C (mg/dL)	51.0 ± 13.5	52.0 ± 13.1	51.8 ± 12.1	49.0 ± 11.3	50.3 ± 13.9	50.1 ± 11.9
Non-HDL-C (mg/dL)	178.1 ± 25.5	144.7 ± 23.6 * °	145.3 ± 26.5 * °	183.6 ± 22.2	187.2 ± 24.3	189.6 ± 24.0
Triglycerides (mg/dL)	114.9 ± 46.2	103.7 ± 53.9	113.5 ± 46.8	110.4 ± 46.3	119.5 ± 71.9	127.9 ± 79.6
Apo B (mg/dL)	106.9 ± 13.6	88.5 ± 14.6 *°	92.1 ± 17.0 * °	110.8 ± 15.8	111.8 ± 15.9	112.4 ± 19.6
TC/HDL-C	4.76 ± 1.22	4.01 ± 1.07 * °	3.97 ± 0.92 * °	4.98 ± 1.19	5.02 ± 1.34	5.05 ± 1.28
LDL-C/HDL-C	3.25 ± 0.99	2.54 ± 0.81 * °	2.49 ± 0.74 * °	3.48 ± 0.93	3.47 ± 1.05	3.46 ± 0.95
FPG (mg/dL)	89.1 ± 11.6	88.5 ± 12.7	88.2 ± 12.5	88.8 ± 11.4	88.7 ± 12.2	87.2 ± 12.2
ALT (U/L)	22.5 ± 8.6	26.3 ± 12.6	27.1 ± 13.8	23.4 ± 10.3	27.2 ± 17.1	26.3 ± 14.6
AST (U/L)	20.9 ± 5.6	23.6 ± 7.6	21.8 ± 5.6	21.7 ± 4.0	21.7 ± 5.5	22.6 ± 4.8
γ-GT (U/L)	22.1 ± 13.6	22.9 ± 16.4	23.2 ± 17.5	21.2 ± 13.1	24.4 ± 25.8	24.3 ± 21.2
SUA (mg/dL)	4.4 ± 1.3	4.1 ± 0.9	4.1 ± 1.0	4.2 ± 0.9	4.2 ± 0.9	4.2 ± 0.9
Serum creatinine (mg/dL)	0.94 ± 0.13	0.95 ± 0.13	0.94 ± 0.11	0.96 ± 0.16	0.99 ± 0.14	0.96 ± 0.15
sGFR (mL/min/1.73 m^2^)	77.3 ± 8.3	77.2 ± 7.9	77.4 ± 7.8	76.1 ± 11.9	77.2 ± 10.9	76.9 ± 9.3
CPK (U/L)	110 ± 65	125 ± 85	112 ± 61	131 ± 77	124 ± 76	124 ± 61

* *p* < 0.01 vs. baseline; ° *p* < 0.01 vs. control. TC = Total Cholesterol, ApoB = Apolipoprotein B, FPG = Fasting Plasma Glucose, ALT = Alanine Aminotranferase, AST = Aspartate Aminotranferase, gGT = γ-glutamyl transferase, SUA = Serum Uric Acid, eGFR = estimated glomerular filtration rate, CPK = creatine phosphokinase.

## References

[1] Bittner V.A. (2019). The New 2019 ACC/AHA Guideline on the Primary Prevention of Cardiovascular Disease. Circulation..

[2] Michos E.D., McEvoy J.W., Blumenthal R.S. (2019). Lipid Management for the Prevention of Atherosclerotic Cardiovascular Disease. N. Engl. J. Med..

[3] Ferraro R.A., Fischer N.M., Xun H., Michos E.D. (2020). Nutrition and physical activity recommendations from the United States and European cardiovascular guidelines: A comparative review. Curr. Opin. Cardiol..

[4] Cicero A.F., Fogacci F., Colletti A. (2017). Food and plant bioactives for reducing cardiometabolic disease risk: An evidence based approach. Food Funct..

[5] Cicero A.F., Colletti A., Bajraktari G., Descamps O., Djuric D.M., Ezhov M., Fras Z., Katsiki N., Langlois M., Latkovskis G. (2017). Lipid-lowering nutraceuticals in clinical practice: Position paper from an International Lipid Expert Panel. Nutr. Rev..

[6] Authors/Task Force Members, ESC Committee for Practice Guidelines (CPG), ESC National Cardiac Societies (2019). 2019 ESC/EAS guidelines for the management of dyslipidaemias: Lipid modification to reduce cardiovascular risk. Atherosclerosis.

[7] Goldstein M.R. (2000). Effects of dietary phytosterols on cholesterol metabolism and atherosclerosis. Am. J. Med..

[8] Cedó L., Farràs M., Lee-Rueckert M., Escolà-Gil J.C. (2019). Molecular Insights into the Mechanisms Underlying the Cholesterol-Lowering Effects of Phytosterols. Curr. Med. Chem..

[9] Ras R.T., Geleijnse J.M., Trautwein E.A. (2014). LDL-cholesterol-lowering effect of plant sterols and stanols across different dose ranges: A meta-analysis of randomised controlled studies. Br. J. Nutr..

[10] Han S., Jiao J., Xu J., Zimmermann D., Actis-Goretta L., Guan L., Zhao Y., Qin L. (2016). Effects of plant stanol or sterol-enriched diets on lipid profiles in patients treated with statins: Systematic review and meta-analysis. Sci. Rep..

[11] Fumeron F., Bard J.M., Lecerf J.M. (2017). Interindividual variability in the cholesterol-lowering effect of supplementation with plant sterols or stanols. Nutr. Rev..

[12] EFSA Panel on Dietetic Products, Nutrition and Allergies (NDA) (2014). Scientific Opinion on the modification of the authorisation of a health claim related to plant sterol esters and lowering blood LDL-cholesterol; high blood LDL-cholesterol is a risk factor in the development of (coronary) heart disease pursuant to Article 14 of Regulation (EC) No 1924/2006, following a request in accordance with Article 19 of Regulation (EC) No 1924/2006. EFSA J..

[13] Cicero A.F., Fogacci F., Banach M. (2019). Red Yeast Rice for Hypercholesterolemia. Methodist Debakey Cardiovasc. J..

[14] Ma J., Li Y., Ye Q., Li J., Hua Y., Ju D., Zhang D., Cooper R., Chang M. (2000). Constituents of red yeast rice, a traditional Chinese food and medicine. J. Agric. Food Chem..

[15] Gerards M.C., Terlou R.J., Yu H., Koks C.H., Gerdes V.E. (2015). Traditional Chinese lipid-lowering agent red yeast rice results in significant LDL reduction but safety is uncertain—A systematic review and meta-analysis. Atherosclerosis.

[16] EFSA (2011). Scientific Opinion on the substantiation of health claims related to monacolin K from red yeast rice and maintenance of normal blood LDL-cholesterol concentrations (ID 1648, 1700) pursuant to Article 13 of Regulation (EC) No 1924/20061; EFSA Panel on Dietetic Products, Nutrition and Allergies (NDA), European Food Safety Authority (EFSA), Parma, Italy. EFSA J..

[17] EFSA (2018). Scientific opinion on the safety of monacolins in red yeast rice. EFSA Panel on Food Additives and Nutrient Sources added to Food (ANS). European Food Safety Authority (EFSA), Parma, Italy. EFSA J..

[18] Fogacci F., Banach M., Mikhailidis D.P., Bruckert E., Toth P.P., Watts G.F., Reiner Ž., Mancini J., Rizzo M., Mitchenko O. (2019). Safety of red yeast rice supplementation: A systematic review and meta-analysis of randomized controlled trials. Pharmacol. Res..

[19] Cicero A.F., Fogacci F., Bove M., Giovannini M., Borghi C. (2019). Three-arm, placebo-controlled, randomized clinical trial evaluating the metabolic effect of a combined nutraceutical containing a bergamot standardized flavonoid extract in dyslipidemic overweight subjects. Phytother. Res..

[20] Nasi M., Patrizi G., Pizzi C., Landolfo M., Boriani G., Dei Cas A., Cicero A.F., Fogacci F., Rapezzi C., Sisca G. (2019). The role of physical activity in individuals with cardiovascular risk factors: An opinion paper from Italian Society of Cardiology-Emilia Romagna-Marche and SIC-Sport. J. Cardiovasc. Med..

[21] Cicero A.F., Derosa G., Pisciotta L., Barbagallo C., SISA-PUFACOL Study Group (2015). Testing the Short-Term Efficacy of a Lipid-Lowering Nutraceutical in the Setting of Clinical Practice: A Multicenter Study. J. Med. Food.

[22] Cicero A.F., Brancaleoni M., Laghi L., Donati F., Mino M. (2005). Antihyperlipidaemic effect of a Monascus purpureus brand dietary supplement on a large sample of subjects at low risk for cardiovascular disease: A pilot study. Complement. Ther. Med..

[23] Demonty I., Ras R.T., van der Knaap H.C., Meijer L., Zock P.L., Geleijnse J.M., Trautwein E.A. (2013). The effect of plant sterols on serum triglyceride concentrations is dependent on baseline concentrations: A pooled analysis of 12 randomised controlled trials. Eur. J. Nutr..

[24] Cicero A.F., Fogacci F., Bove M., Veronesi M., Rizzo M., Giovannini M., Borghi C. (2017). Short-Term Effects of a Combined Nutraceutical on Lipid Level, Fatty Liver Biomarkers, Hemodynamic Parameters, and Estimated Cardiovascular Disease Risk: A Double-Blind, Placebo-Controlled Randomized Clinical Trial. Adv. Ther..

[25] Cicero A.F., Fogacci F., Morbini M., Colletti A., Bove M., Veronesi M., Giovannini M., Borghi C. (2017). Nutraceutical Effects on Glucose and Lipid Metabolism in Patients with Impaired Fasting Glucose: A Pilot, Double-Blind, Placebo-Controlled, Randomized Clinical Trial on a Combined Product. High. Blood Press Cardiovasc. Prev..

[26] Palmieri L., Donfrancesco C., Giampaoli S., Trojani M., Panico S., Vanuzzo D., Pilotto L., Cesana G., Ferrario M., Chiodini P. (2006). Favorable cardiovascular risk profile and 10-year coronary heart disease incidence in women and men: Results from the Progetto CUORE. Eur. J. Cardiovasc. Prev. Rehabil..

[27] Cicero A.F., Colletti A. (2016). Combinations of phytomedicines with different lipid lowering activity for dyslipidemia management: The available clinical data. Phytomedicine.

[28] Rosato V., Temple N.J., La Vecchia C., Castellan G., Tavani A., Guercio V. (2019). Mediterranean diet and cardiovascular disease: A systematic review and meta-analysis of observational studies. Eur. J. Nutr..

[29] Cholesterol Treatment Trialists’ (CTT) Collaboration (2010). Efficacy and safety of more intensive lowering of LDL cholesterol: A meta-analysis of data from 170000 participants in 26 randomised trials. Lancet.

[30] Cicero A.F., Fogacci F., Rosticci M., Parini A., Giovannini M., Veronesi M., D’Addato S., Borghi C. (2017). Effect of a short-term dietary supplementation with phytosterols, red yeast rice or both on lipid pattern in moderately hypercholesterolemic subjects: A three-arm, double-blind, randomized clinical trial. Nutr. Metab..

[31] Baila-Rueda L., Pérez-Ruiz M.R., Jarauta E., Tejedor M.T., Mateo-Gallego R., Lamiquiz-Moneo I., de Castro-Orós I., Cenarro A., Civeira F. (2016). Cosegregation of serum cholesterol with cholesterol intestinal absorption markers in families with primary hypercholesterolemia without mutations in LDLR, APOB, PCSK9 and APOE genes. Atherosclerosis.

[32] EFSA Panel on Dietetic Products, Nutrition and Allergies (NDA) (2009). Scientific Opinion on the substantiation of health claims related to niacin and energy-yielding metabolism (ID 43, 49, 54), function of the nervous system (ID 44, 53), maintenance of the skin and mucous membranes (ID 45, 48, 50, 52), maintenance of normal LDL-cholesterol, HDL-cholesterol and triglyceride concentrations (ID 46), maintenance of bone (ID 50), maintenance of teeth (ID 50), maintenance of hair (ID 50, 2875) and maintenance of nails (ID 50, 2875) pursuant to Article 13 of Regulation (EC) No 1924/2006 on request from European Commission. EFSA J..

